# Overcoming anticancer resistance by photodynamic therapy-related efflux pump deactivation and ultrasound-mediated improved drug delivery efficiency

**DOI:** 10.1186/s40580-020-00241-8

**Published:** 2020-09-08

**Authors:** Doyeon Kim, Suhyun Park, Hongkeun Yoo, Suhyeon Park, Jeewon Kim, Kyuhee Yum, Kwangmeyung Kim, Hyuncheol Kim

**Affiliations:** 1 Department of Chemical & Biomolecular Engineering, Sogang University, 35 Baekbeom-ro, Mapo-gu, Seoul, 04107 Republic of Korea; 2 KU-KIST Graduate School of Converging Science and Technology, Korea University, 145 Anam-ro, Seongbuk-gu, Seoul, 02841 Republic of Korea; 3 Biomedical Research Institute, Korea Institute of Science and Technology (KIST), Seoul, 02792 Republic of Korea; 4 Department of Biomedical Engineering, Sogang University, 35 Baekbeom-ro, Mapo-gu, Seoul, 04107 Republic of Korea

**Keywords:** Multidrug resistance, Nanomedicine, Ultrasound, Microbubble, Sonoporation, Efflux pump, Side population

## Abstract

One of the major obstacles to successful chemotherapy is multi-drug resistance (MDR). A multi-drug resistant cancerous cell abnormally overexpresses membrane transporters that pump anticancer drugs out of the cell, resulting in low anticancer drug delivery efficiency. To overcome the limitation, many attempts have been performed to inhibit the abilities of efflux receptors chemically or genetically or to increase the delivery efficiency of anticancer drugs. However, the results have not yet been satisfactory. In this study, we developed nanoparticle-microbubble complexes (DOX-NPs/Ce6-MBs) by conjugating doxorubicin loaded human serum albumin nanoparticles (DOX-NPs) onto the surface of Chlorin e6 encapsulated microbubbles (Ce6-MBs) in order to maximize anticancer efficiency by overcoming MDR. Under the ultrasound irradiation, DOX-NPs and Ce6 encapsulating self-assembled liposomes or micelles were effectively delivered into the cells due to the sonoporation effect caused by the microbubble cavitation. At the same time, reactive oxygen (ROS) generated from intracellularly delivered Ce6 by laser irradiation arrested the activity of ABCG2 efflux receptor overexpressed in doxorubicin-resistant breast cancer cells (MCF-7/ADR), resulting in increased the chemotherapy efficacy. In addition, the total number of side population cells that exhibit the properties of cancer stem-like cells were also reduced by the combination of photodynamic therapy and chemotherapy. In conclusion, DOX-NPs/Ce6-MBs will provide a platform for simultaneously overcoming MDR and increasing drug delivery and therefore, treatment efficiency, under ultrasound irradiation.

## Introduction

Multi-drug resistance (MDR), which is the phenomenon of showing resistance to anticancer drugs, is one of the major barriers in chemotherapy [1]. MDR in cancer cells is often related to the overexpression of efflux pump receptors, such as P-glycoprotein (P-gp), which is a family of ATP-binding cassette (ABC) transporter proteins, and responsible for pumping out exogenous materials from cells [2]. As P-gp actively pumps drugs out of cancer cells, the intracellular concentration of the chemotherapeutic agents dramatically reduces. Among multidrug resistant cancer cells with ABC efflux pump receptors, some cells can be isolated by their capacity to efflux Hoechst 33342 dye. This particular cell population is called the side population (SP) because flow-cytometry analysis places these cells on the side of the main population. SP cells have been shown to possess stem cell-like characteristics, such as self-renewal, differentiation potential, multidrug resistance, and apoptosis resistance [3]. Cells with overexpression of efflux pump receptors and side population characteristics contribute to the multidrug resistance and high proliferation, making it difficult for chemotherapy. Many studies have been reported to overcome these multi-drug resistances. One of the representative studies inhibited efflux pump receptors using MDR modulators, such as verapamil [4], gallopamil [5], and tariquidar [6], to reduce efflux pump activity. However, the inhibitors were toxic, and they also inhibited the intracellular influx of co-delivered anticancer drugs [7, 8]. Another reported study to overcome multi-drug resistance was to deliver large amounts of chemotherapeutic agents with nanomedicines intracellularly beyond the efflux capacity of cancer cells [9]. Various nanoparticles composed of lipids, polymers or proteins have been investigated as possible carriers to deliver chemotherapeutic agents into cells [10]. However, the delivery efficiency of nanoparticles is still lower than expected, not only because the endocytosis of nanoparticles leads to endosomal entrapment or lysosomal degradation, but also because the MDR efflux receptor pumps intracellularly delivered drugs out of the cells [11]. Also, when nanoparticles are used as a co-delivery system, the loading efficiency can be decreased by the steric hindrance between two drugs. Therefore, a new strategy to overcome the limitations of chemotherapy due to the overexpression of efflux receptors is required.

**Scheme 1:**
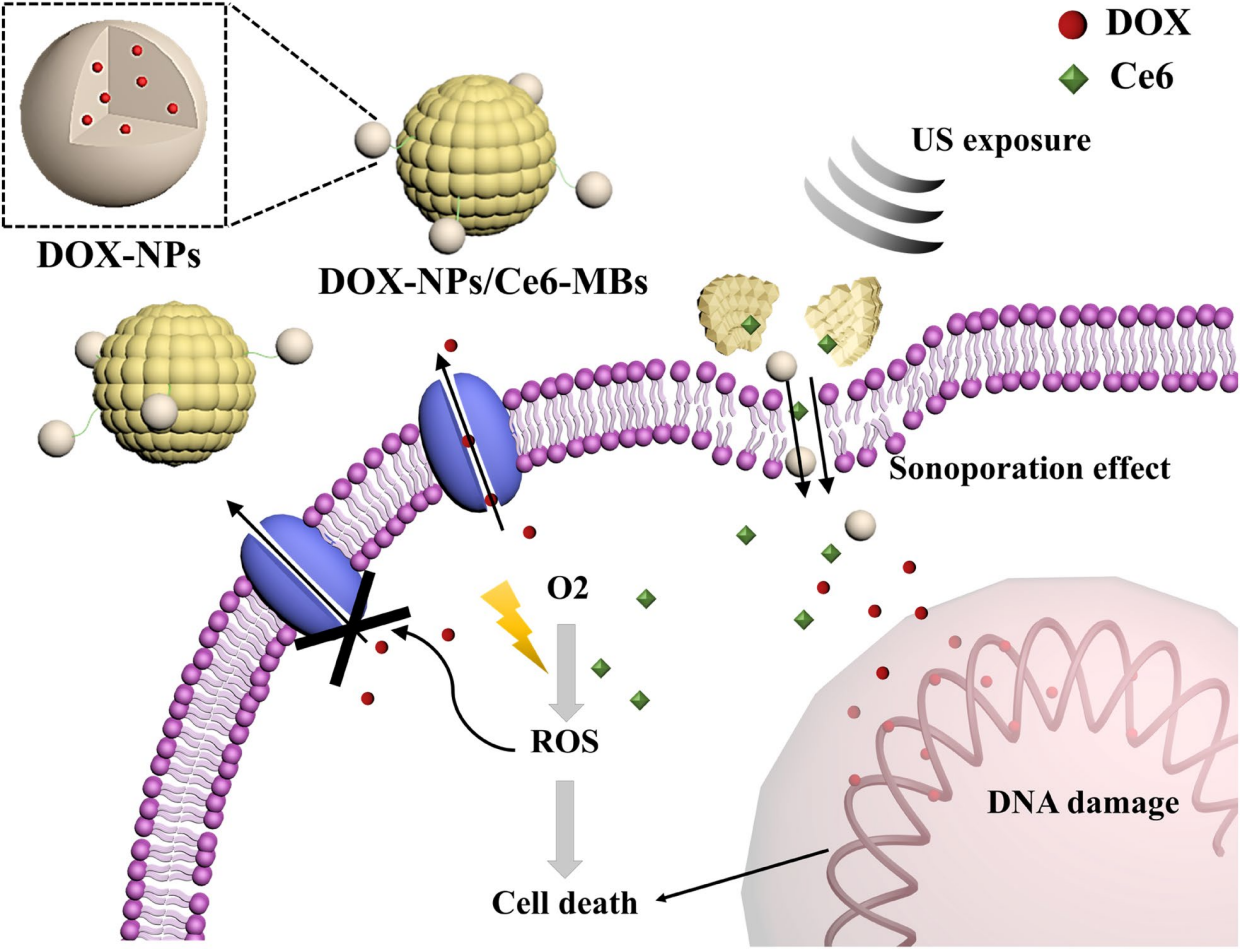
Schematic illustration of this study demonstrating mechanisms to overcome anticancer resistance of MCF-7/ADR cells using DOX-NPs/Ce6-MBs with US and laser irradiation in MCF-7/ADR cells

In this study, we developed doxorubicin-loaded nanoparticles-Chlorin e6-encapsulating microbubbles complex (DOX-NPs/Ce6-MBs) to overcome anticancer multi-drug resistance by effectively delivering anticancer drugs intracellularly beyond the efflux capacity of MDR cancer cells (Scheme 1). Microbubbles, composed of phospholipids, are used broadly as a drug delivery system as well as an ultrasound (US) contrast agent [12]. When exposed to US, the microbubbles oscillate and eventually cavitate, producing a jet stream that creates temporary pores which increase the permeability of the cell membrane. This phenomenon is called the sonoporation effect and it can be applied to effectively deliver doxorubicin-loaded nanoparticles (DOX-NPs) into cancer cells [1, 13]. Also, after the cavitation, Chlorin e6 (Ce6)-encapsulating microbubbles (Ce6-MB) are self-assembled into Ce6-encapsulating liposomes (or micelles) and delivered into the cells through the temporary pores [14]. When irradiated with a laser, the intracellularly delivered Ce6 produces reactive oxygen species (ROS), which in turn damages macromolecules in the cell membrane, including the efflux pump receptors [15, 16]. Photodynamic therapy (PDT) for multi-drug resistant cancer cells interferes with the function of over-expressed efflux pump receptors, improving the efficacy of anticancer drugs delivered intracellularly. In this study, we experimentally determined the synergistic effect of combined PDT and US-mediated chemotherapy to overcome MDR in vitro using DOX-NPs/Ce6-MBs complexes exposed to both US and laser radiation. Our results showed that the DOX-NPs/Ce6-MBs complex system using PDT and US has a high potential as a novel carrier for MDR cells by overcoming limited drug delivery efficiency due to over-expressed efflux pump receptors.

## Methods

### Materials

Human serum albumin (HSA), 2-iminothiolane hydrochloride (Traut’s reagent, 2-IT), doxorubicin hydrochloride (DOX), dimethyl sulfoxide (DMSO), methanol, and chloroform were purchased from Sigma Aldrich (St. Louis, MO, USA). Two types of phospholipids, 1,2-distearoyl-sn-glycero-3-phosphocholine (DSPC) and 1,2-distearoyl-sn-glycero-3-phosphoethanolamine-*N*-[succinyl(polyethylene glycol)-2000] (DSPE-PEG2k-NHS), were purchased from Nanocs Inc. (Boston, MA, USA). Chlorin e6 was purchased from Santa Cruz Biotechnology (Santa Cruz, CA, USA). RPMI 1640, fetal bovine serum (FBS), and antibiotic–antimycotic (AA) were purchased from Gibco (Waltham, MA, USA). The adriamycin resistant breast cancer cell line (MCF-7/ADR) was donated from Dr. Kwang Meyung Kim (Korea Institute of Science and Technology, Seoul, Korea).

### Preparation of DOX-NPs/Ce6-MBs

First, 40 mg/mL of human serum albumin was dissolved in distilled water and the pH was adjusted to 8.5 using 0.2 N NaOH. For the thiolation of HSA, albumin solution was mixed with 2 mg/mL 2-iminothiolane hydrochloride (2-IT) in a 1:1 volume ratio and incubated for 1 h at room temperature (RT). The thiolated HSA (tHSA) solution was centrifuged at 4000 rpm for 10 min using an Amicon Ultra-30 kDa Centricon (Millipore, Billerica, MA, USA) to remove unreacted 2-IT. Then, 10 mg/mL doxorubicin (DOX) dissolved in distilled water was mixed with the tHSA solution in a 1:9 volume ratio. Ethanol was added dropwise in 1 mL/min until the solution became turbid and stirred overnight at RT. After the ethanol was almost evaporated, the tHSA solution was centrifuged at 13,200 rpm for 10 min to harvest nano-sized DOX-NPs.

Microbubbles were produced by mixing DSPC and DSPE-PEG2k-NHS in chloroform in a 9:1 molar ratio. Then, 0.5 mg/mL of Chlorin e6 dissolved in methanol was added to the lipid mixture and dried overnight under a vacuum. Dried lipid and Ce6 were dissolved in 0.5 mg/mL phosphate-buffered saline (PBS), and sonicated using a bath sonicator and incubated in hot water (over 55 °C). Then, the vial was filled with perfluoropropane gas and mixed for 45 s using VialMix™ to form the Ce6-MBs. To complete the fabrication of DOX-NPs/Ce6-MBs, DOX-tHSA-NPs and Ce6-MBs were mixed and incubated at RT for at least 1 h. The conjugation of DOX-NPs on the surface of Ce6-MBs was produced by an amide bond between the amine group of the DOX-tHSA-NPs and the NHS group of the Ce6-MBs.

### Characterizations of DOX-NPs, Ce6-MBs, and DOX-NPs/Ce6-MBs

The size distributions of the DOX-NPs, MBs, and Ce6-MBs were analyzed by dynamic light scattering (Zetasizer Nano ZS, Malvern Instruments, Herrenberg, Germany). The in vitro release rate of DOX from the DOX-NPs was measured using high-performance liquid chromatography (HPLC) (YL9150, Younglin Anyang, South Korea). Each group was loaded into the dialysis membrane (Spectra/Por^®^ 7, MWCO 2 kD) and then the drug-loaded dialysis membrane was placed in 10 mL of PBS (in a 15 mL tube). Dialysis membranes were maintained in a 37 °C shaking incubator. At predetermined time points, 10 mL of the PBS buffer was replaced with fresh buffer. Fluorescence imaging of the DOX-NPs/Ce6-MBs was conducted by confocal laser scanning microscopy (TCS SP8, Leica, Germany). The cavitation confdition of the microbubble was 1 MHz-US pressure waves (Sonoplus 490, Enraf–Nonius B.V., Rotterdam, Netherlands) with an intensity of 0.2 W/cm^2^ and a duty cycle of 50%.

### Confirmation of the expression of drug efflux pump receptors

The expression levels of efflux pump receptor mRNAs in the MCF-7 cells and MCF-7/ADR cells were analyzed using reverse transcription-polymerase chain reaction (RT-PCR) and real-time PCR. Aliquots of 1 × 10^6^ cells in 1 mL serum-free RPMI 1640 were placed in each well of a 6-well plate and incubated at 37 °C for 24 h. The total RNA was obtained using a RNeasy Mini Kit purchased from Qiagen (Venlo, Netherlands) and cDNA was synthesized using a High Capacity RNA-to-cDNA Kit purchased from Thermo Fisher Scientific (Waltham, MA, USA). RT-PCR was performed using AccuPower PCR PreMix purchased from BIONEER (Daejeon, Korea) and LightCycler FastStart DNA Master^PLUS^ SYBR Green purchased from Roche (Basel, Switzerland) was used for real-time PCR. ABCB1 (P-glycoprotein, P-gp), ABCC1 (multidrug resistance-associated protein 1, MRP1), and ABCG2 (ATP binding cassette subfamily G member 2) primers were purchased from BIONEER (Daejeon, Korea). Fluorescence imaging of P-glycoprotein was detected using rabbit anti-P-glycoprotein antibody and Alexa Fluor 555 goat anti-rabbit antibody purchased from Abcam (Cambridge, UK) by confocal laser scanning microscopy.

### Determination of the intracellular uptake and retention of DOX and Ce6

The intracellular uptake of DOX and Ce6 was determined by confocal laser scanning microscopy. Aliquots of 2 × 10^5^ MCF-7/ADR cells in 1 mL RPMI 1640 were seeded into each well of a collagen-coated 12-well plate and incubated at 37 °C for 24 h. US was irradiated immediately after treatment with DOX-NPs, Ce6-MBs, and DOX-NPs/Ce6-MBs (0.2 W/cm^2^, 50% duty cycle, 30 s/well) and the cells were incubated for an additional 3 h. Each group was treated with 2 mg/mL DOX and 1 µg/mL Ce6. After another incubation, the cells were washed with cold DPBS (Dulbecco’s Phosphate-Buffered Saline) and fixed with 4% paraformaldehyde. The cells were mounted using a mounting solution and stained with DAPI (4′,6-diamidino-2-phenylindole) (Vectashield, Vector Laboratories. Inc., Burlingame, CA, USA).

To verify the intracellular retention of DOX, each group was treated with DOX-NPs/Ce6-MBs. The groups included control (no treatment), verapamil-treated (positive control), and laser-treated groups. Each group was treated with DOX-NPs/Ce6-MBs and US and incubated for 3 h. After 3 h, each group was washed with PBS and incubated further for different times (in 0.5 h increments for confocal microscopy and l h increments for flow cytometry). At each time point, the cells were fixed with 4% paraformaldehyde and examined using confocal microscopy and flow cytometry.

### Measurement of ROS generation

For the detection of ROS generation by the DOX-NPs, Ce6-MBs, and DOX-NPs/Ce6-MBs, the DCFDA kit (Cellular Reactive Oxygen Species Detection Assay Kit, Abcam, Cambridge, UK) was used. MCF-7/ADR cells in 100 µL RPMI 1640 were cultured in 96-well plates to over 80% density and incubated overnight. Then, all groups were changed to DCFDA solution, incubated for 45 min at 37 °C, and the DOX-NPs/Ce6-MBs were treated with 2 mg/mL DOX and 1 µg/mL Ce6. The cells were immediately irradiated with US using the same conditions as in the cellular uptake experiment, incubated for 3 h, and laser-irradiated (671 nm wavelength, 1.0 J/cm^2^, 100 mW). The intracellular ROS levels were measured at 488 nm by a microplate reader (Bio-Tek, Winooski, VT, USA).

### Side Population assay

For detection of the side population, 3 × 10^5^ MCF-7/ADR cells in 1 mL RPMI 1640 were cultured in 6-well plates. The cells were incubated at 37 °C for 90 min with Hoechst 33342 dye (Sigma Aldrich) alone, or with 50 µM verapamil (Sigma Aldrich). The cells were washed with DPBS twice, centrifuged, and re-suspended in 2% FBS/PBS solution. The cells were analyzed with image cytometry based on the intracellular levels of Hoechst 33342 dye.

### In vitro cell viability assay

To confirm the cytotoxic effect of the DOX-NPs/Ce6-MBs with laser via sonoporation an MTT assay was performed in the MCF-7/ADR cells. The cells (1 × 10^4^) were seeded in each 96-well plate and cultured at 37 °C for 24 h. The groups used in the experiment were negative control (non-treated), free DOX, DOX-NPs, and DOX-NPs/Ce6-MBs (N = 6) which were further divided into irradiation with US (0.3 W/cm^2^, 50% duty cycle, 10 s/well) and laser (671 nm wavelength, 1.0 J/cm^2^, 50 mW) groups. After a 3 h incubation, all groups were changed to fresh media and cultured for a further 48 h. To perform the MTT assay, MTT solution (0.5 mg/mL) was added to each well and the cells were incubated at 37 °C for 2 h. Then, 100 µL of DMSO was added to dissolve the formazan crystals in the living cells. The absorbance intensity was measured at 570 nm by a microplate reader (Bio-Tek).

### Statistical analysis

All experimental data are presented as the mean ± standard error of at least three independent experiments. Statistical analysis was performed using Student’s t-test and one-way ANOVA were used for multiple comparisons. A p-value less than 0.05 was considered statistically significant.

## Results

### Characteristics of DOX-NPs/Ce6-MBs complexes

**Fig. 1:**
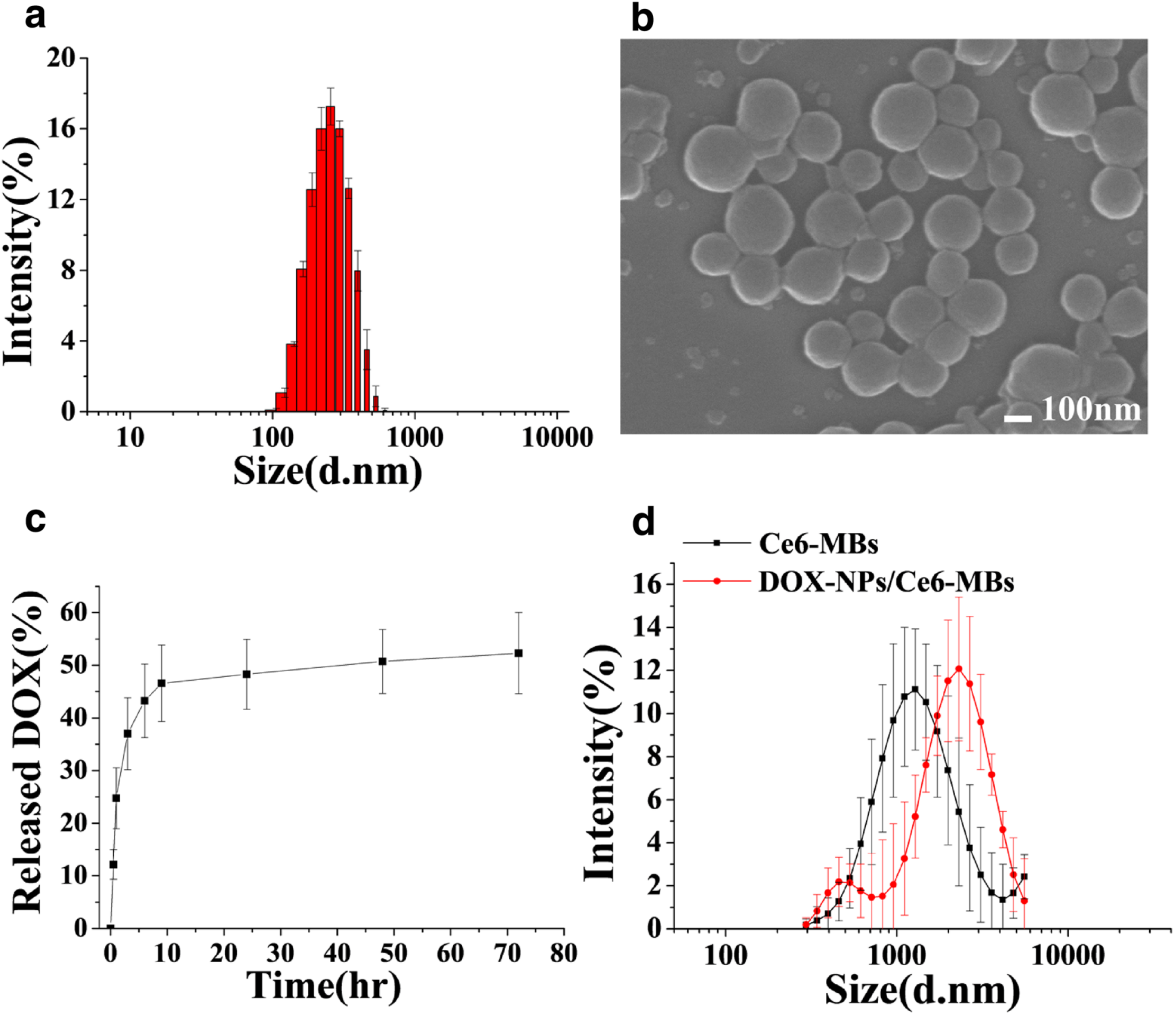
Characteristics of the DOX-NPs/Ce6-MBs complex. **a** Size distribution of the DOX-NPs. **b** Scanning electron microscopy (SEM) image of DOX-NPs. Scale bar represents 100 nm. **c** In vitro release profile of DOX from DOX-NPs. **d** Size distribution of the Ce6-MBs and DOX-NPs/Ce6-MBs

The DOX-NPs/Ce6-MBs complex was developed by conjugating doxorubicin-encapsulating human serum albumin nanoparticles (DOX-NPs) onto the surface of Chlorin e6-encapsulating microbubbles (Ce6-MBs). The DOX-NPs demonstrates an average size distribution of 233.80 ± 0.63 nm (Fig. 1a). Scanning electron microscopy images of the DOX-NPs showed uniform spherically shaped nanoparticles (Fig. 1b). The anticancer drug, doxorubicin (DOX), released from the DOX-NPs was confirmed by in vitro release assay results (Fig. 1c). In the first 8 h, DOX-NPs released about 36.3% of the encapsulated DOX and after 80 h, they released about 51.4% of the encapsulated DOX totally. To validate the conjugation of DOX-NPs onto the surface of the Ce6-MBs, an increase in the size distribution was confirmed (Fig. 1d). The size of Ce6-MBs increased from 1.30 ± 0.13 to 1.66 ± 0.39 µm after conjugation of the DOX-NPs onto the surface of the Ce6-MBs.

### Expression of drug efflux receptors in MCF-7/ADR cell line

**Fig. 2:**
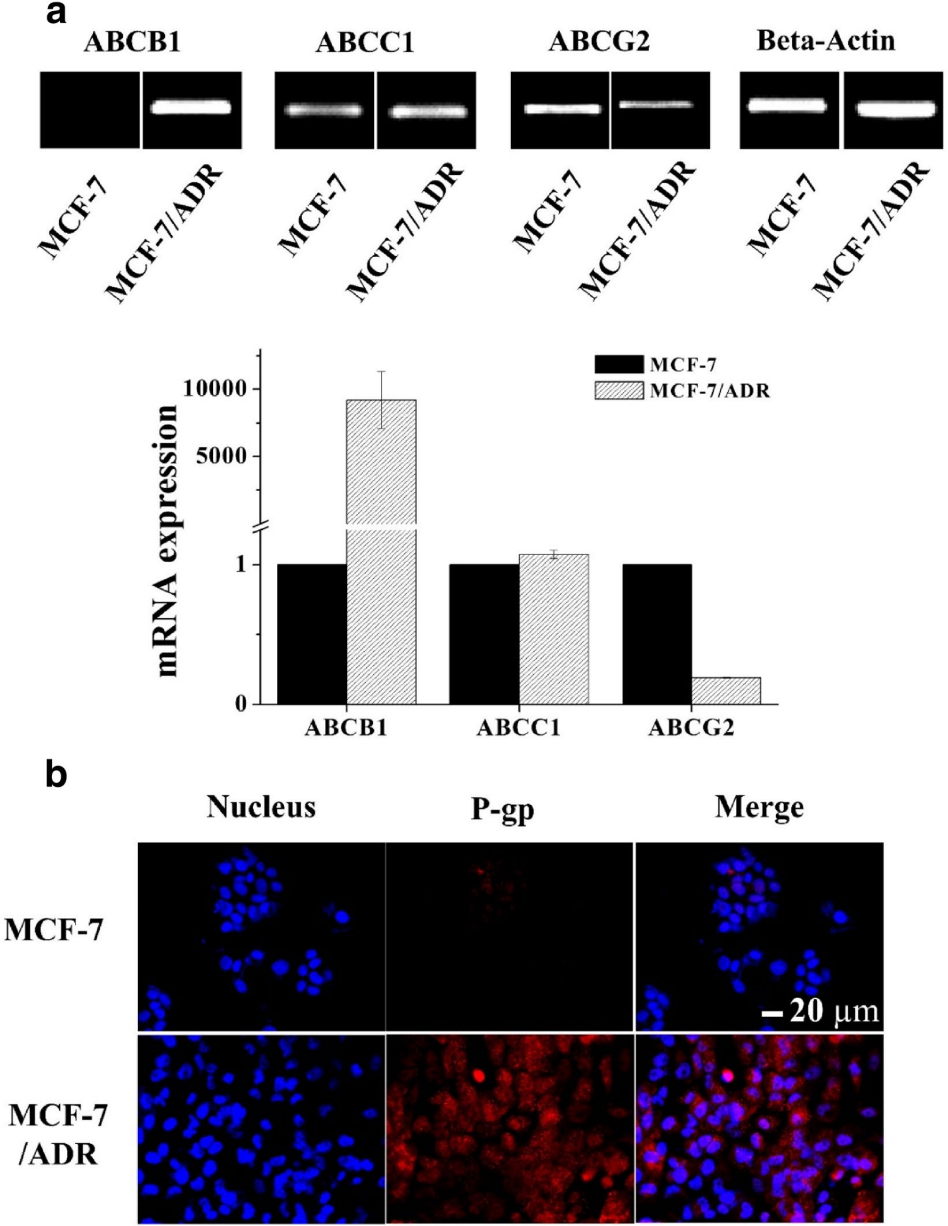
Confirmation of efflux pump receptor expression in MCF-7/ADR cells. **a** Qualitative and quantitative mRNA expression analysis of three types of efflux pump receptors in MCF-7 and MCF-7/ADR cells by reverse transcriptase (RT)-PCR and by real-time PCR, respectively. **b** Immunocytochemical analysis of the expression of P-glycoprotein receptor in MCF-7 and MCF-7/ADR cells (scale bar = 20 µm)

The mRNA expression levels of ABCB1, ABCC1, and ABCG2, known as the drug efflux receptors, in MCF-7/ADR cells, which show resistance to doxorubicin anticancer drug, were compared with those in MCF-7 cells (Fig. 2). The expression level of ABCC1 efflux receptor did not differ between the two cell lines. ABCG2 efflux receptor was found to be slightly more expressed in MCF-7 cells. In contrast, ABCB1 efflux receptor was found to be expressed around 10,000 times more in MCF-7/ADR cells than MCF-7 cells (Fig. 2a). The quantification of mRNA expression by real time-PCR in the MCF-7/ADR and MCF-7 cells showed the same tendency as in Fig. 2a. Immunocytochemical analysis was performed to compare the protein expression levels of efflux receptors (Fig. 2b), demonstrating that the MCF-7/ADR cell line expressed significantly higher P-glycoprotein, also known as ABCB1, than the MCF-7 cells. The ABCB1 efflux receptor is known to selectively pump out doxorubicin anticancer drug [17].

**Fig. 3:**
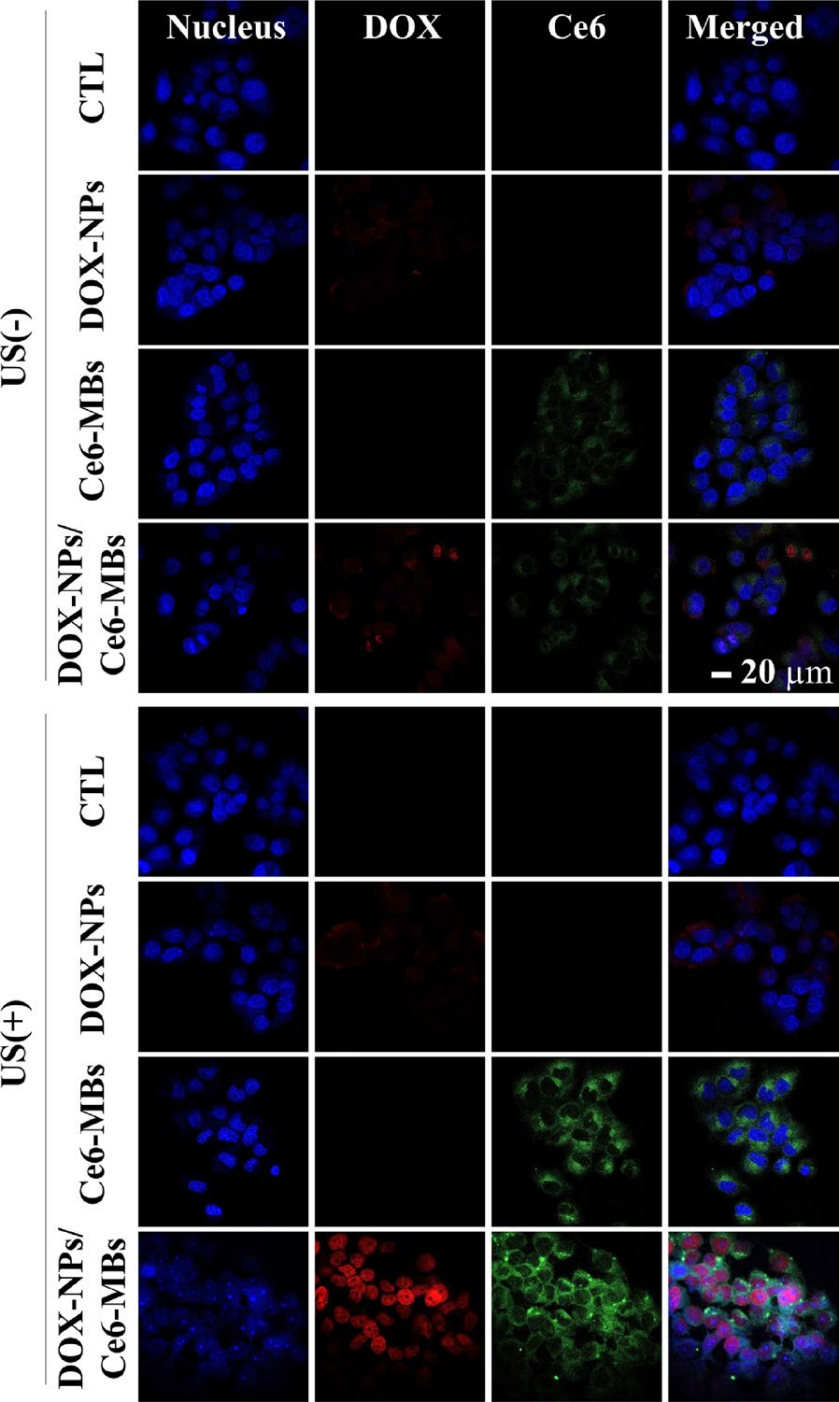
Confocal microscopy images showing intracellular uptake of DOX and Ce6 in MCF-7/ADR cells with or without US irradiation. The blue color represents cell nuclei. Red and green channels represent DOX and Ce6, respectively (scale bar = 20 µm.)

### Intracellular uptake of DOX in MCF-7/ADR cells

The intracellular delivery of DOX and Ce6 was investigated for MCF-7/ADR cells to verify whether the delivery efficiency of each drug was improved according to the ultrasound irradiation (Fig. 3). Figure 3 demonstrates that the intracellular uptake of DOX and Ce6 increased significantly with exposure to US (8th row) compared to without US (4th row). DOX accumulated mainly in the nucleus and Ce6 was taken up in the cytoplasm. Moreover, since the fluorescence of DOX can be detected both on free and NPs-bounded form, the intracellular uptake image shows the intracellular distribution of free DOX and DOX-NPs. The red signals in the cell nucleus and in the cytoplasm indicate free DOX and DOX-NPs, respectively. Figure 1c shows the in vitro release rate of DOX in fresh PBS media. In this case, 20–30% of encapsulated DOX was released after 3 h and about 50% of DOX was not released from the NPs for 70 h. However, since DOX-NPs were delivered intracellularly and degraded enzymatically inside the cells, intracellularly delivered DOX-NPs are expected to release the encapsulated DOX faster than in fresh PBS. The delivery efficiency of the groups treated with DOX-NPs was not different with (6^th^ row) or without US irradiation (3rd row), demonstrating that US itself did not affect intracellular uptake efficiency of DOX-NPs. On the other hand, the intracellular uptake efficiency of Ce6 was increased in US irradiated Ce6-MBs treated group (7th row) compared to the US non-irradiated Ce6-MBs treated group (3rd row), which indicates that US irradiation induced the sonoporation effect due to the cavitation of microbubbles. The sonoporation effect generates jet-streaming, resulting the formation of temporary pores in cell membranes. Nanoparticles are directly delivered through temporary pores in cell membrane, not by endocytosis, which significantly enhances drug delivery efficiency. In addition, microbubbles cavitated by US can be self-assembled into micelles or liposomes [14], thus, using US irradiation, the Ce6-MBs were reassembled into micelles or liposomes and effectively localized into the cells. Overall, Fig. 3 demonstrates that US irradiation delivers both DOX and Ce6 intracellularly much better that those without US irradiation.

### ROS generation of DOX-NPs/Ce6-MBs at the laser irradiation

**Fig. 4:**
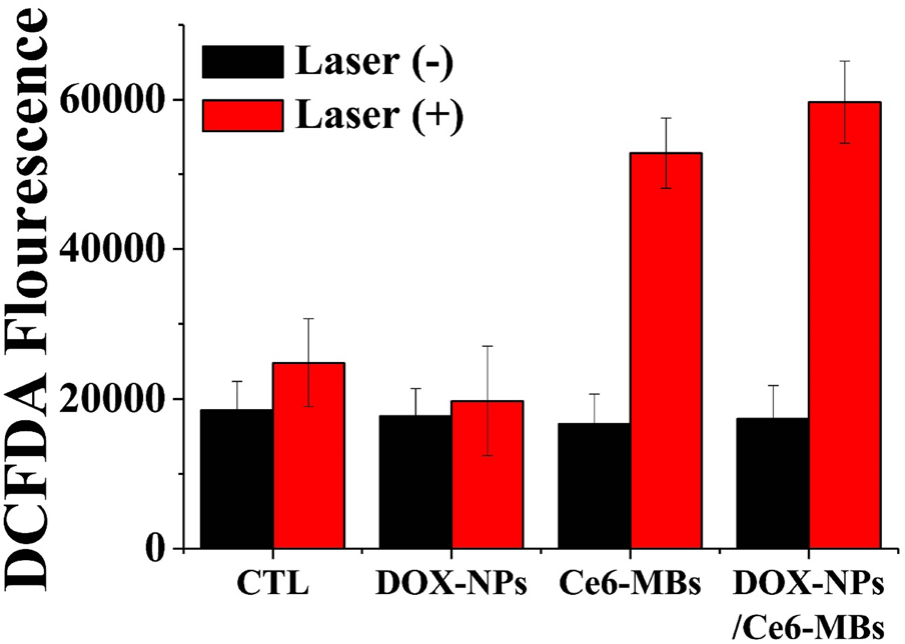
Intracellular ROS generation. The reactive oxygen species (ROS) generation in MCF-7/ADR cells was determined with or without laser irradiation after DOX-NPs/Ce6-MBs treatment with US irradiation (0.2 W/cm^2^, 50% duty cycle, 30 s)

When the laser was irradiated to intracellularly delivered Ce6, the effective ROS generation was confirmed using the DCFDA ROS detection assay (Fig. 4). In the control group treated with PBS (Fig. 4, CTL) and the DOX-NPs alone treated group (Fig. 4, DOX-NPs), there was no statistical difference in ROS generation with or without laser irradiation. Whereas, in the group treated with Ce6-MBs and irradiated with laser, the ROS generation was significantly increased compared to the group treated Ce6-MBs without laser. With laser irradiation, the Ce6-MBs or DOX-NPs/Ce6-MBs treated group showed about a 3.17 or a 3.44-fold increase in ROS generation, respectively, compared to groups without laser irradiation. Figure 4 demonstrate significant high-level ROS generation in the laser irradiation to Ce6 in cells under the exposure of US.

### Intracellular retention of DOX

**Fig. 5:**
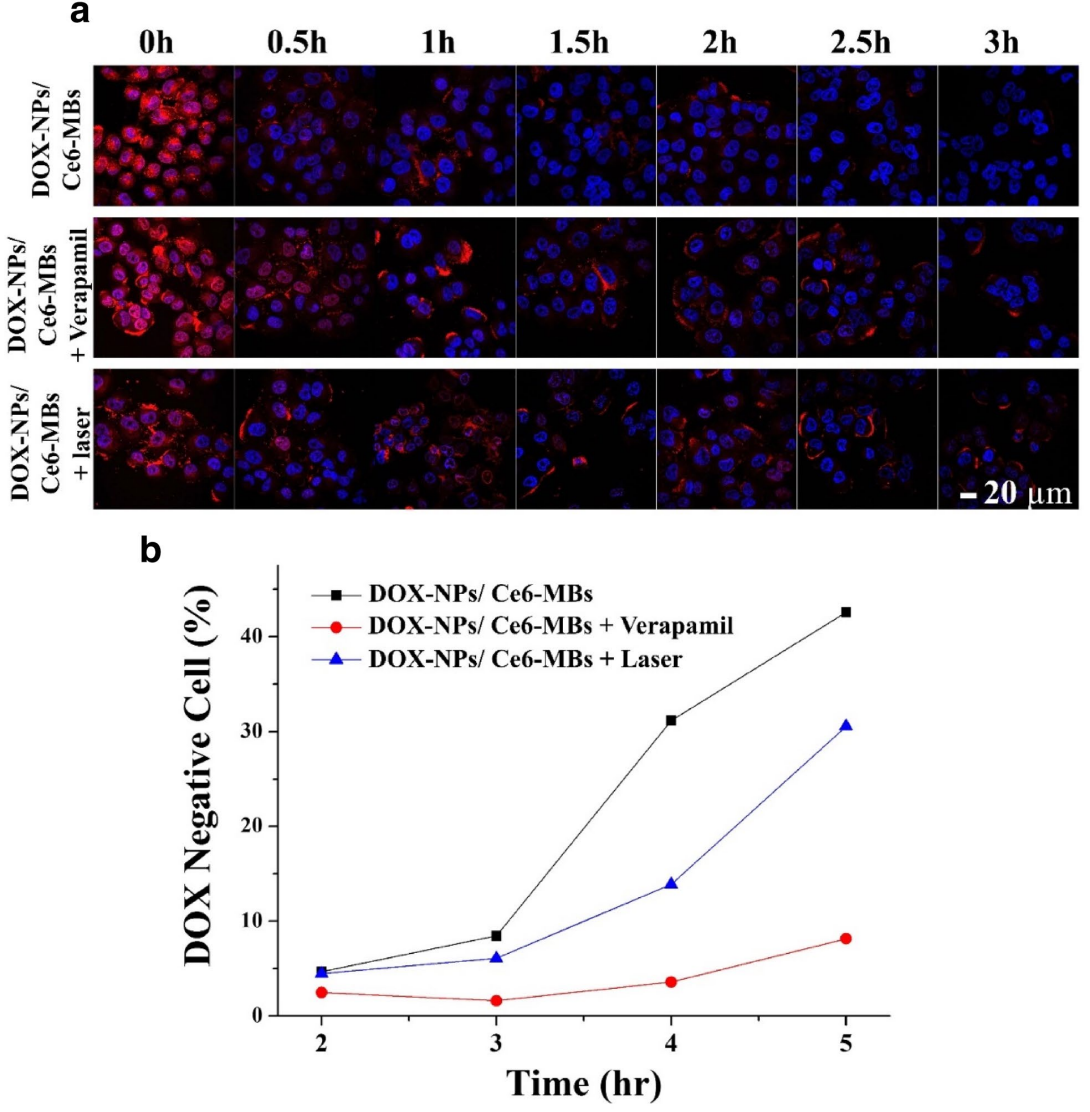
Intracellular retention of DOX in MCF-7/ADR cells. **a** Fluorescence images showing DOX retention inside MCF-7/ADR cells after DOX-NPs/Ce6-MBs treatment and subsequent US irradiation (0.2 W/cm^2^, 50% duty cycle, 30 s) every 30 min (scale bar = 20 µm). **b** Quantitative flow cytometry data showing MCF-7/ADR cell percentages without DOX because of pumping out

To determine the extent to which efflux receptor activity was decreased by ROS generation by laser irradiation in the DOX/NPs/Ce6-MBs treated group under the exposure of US, the intracellular DOX retention was determined by confocal microscopy and flow cytometry (Fig. 5). In Fig. 5a, all group were subjected to DOX-NPs/Ce6-MBs, then irradiated with US and incubated for 3 h. After 3 h, every group was washed with PBS and incubated further for different times (in 0.5 h intervals). The 0 h (1st column) indicates the intracellular uptake level of DOX immediately after the 3 h incubation. At this time, DOX was well-dispersed in all groups, consistent with the cellular uptake data (Fig. 3). The subsequent changes in intracellular DOX concentrations at 30 min intervals showed that DOX remained in the cells longer in the verapamil-treated (positive control) and laser-treated groups than DOX-NPs/Ce6-MBs alone-treated group (negative control). After 3 h, DOX was observed only in the verapamil-treated and laser-treated groups. At 0 h, most of the DOX was in the nucleus, but as time passed, DOX was found in the outer part of the nucleus and in the cytoplasm, demonstrating that the MDR efflux receptors, which present in the cell membrane, pump DOX out from the cells. To quantitatively confirm the DOX retention, the percentage of DOX-negative cells (cells in which DOX fluorescence cannot be detected) were measured by flow cytometry (Fig. 5b). All groups were treated with DOX-NPs/Ce6-MBs and US irradiation and incubated for 3 h. After 3 h, each group was washed with PBS and incubated for 0, 1, 2, and 3 h. At the 2 h point, with laser exposure, DOX in the DOX-NPs/Ce6-MBs treated group with US irradiation (DOX-NPs/Ce6-MBs + laser) remained 2.24 times more than that in the negative control group (DOX-NPs/Ce6-MBs). Figure 4 shows ROS generation according to whether laser was irradiated in DOX-NPs/Ce6-MBs treated group. Figure 5 demonstrates that DOX was lasted intracellularly for a long period of time in the DOX-NPs/Ce6-MBs treated group with US irradiation. These results indicate indirectly that the generated ROS lower the efflux receptor function and prevents the effective pumping out of DOX.

### Side population assay of DOX-NPs/Ce6-MBs-treated cells

**Fig. 6:**
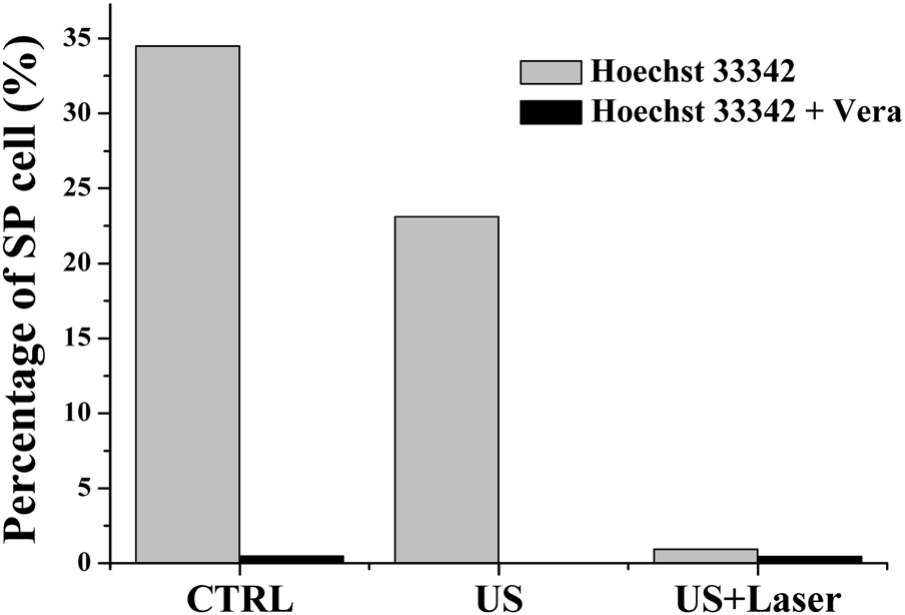
Side population analysis. Image cytometry data showing percentage of side population MCF-7/ADR cells after treatment with DOX-NPs/Ce6-MBs, followed by US or US + Laser irradiation

Side population (SP) cells are characterized by dye exclusion ability mediated by ABC-transporters, ABCG2 [18] and have the characteristics of cancer stem-like cells such as chemotherapy resistance. The subpopulation cells, pumping Hoechst 33342 dye out by ABC pump-mediated transport system, are sorted and detected by flow cytometry (Fig. 6) [19]. Hoechst 33342^low/neg^ cells (SP fraction) were sorted from the MCF-7/ADR cells by image cytometry. In the control group, treated only with DOX-NPs/Ce6-MBs, the SP (Hoechst 33342^low/neg^) fraction accounted for 34.5%. After treatment with verapamil, which is an inhibitor of ABC transporters, the SP fraction of control group significantly decreased to 0.48%. The SP fraction in the US-treated group was 23.1%. Ce6, which can also act as a sonodynamic sensitizer, might generate ROS under the exposure of US by the same mechanism as PDT and slightly arrested the efflux receptor [20]. However, with laser exposure, the SP fraction dramatically decreased to 0.93%, indicating that the most of SP cells were eliminated due to the PDT effect. Since PDT treatment can modulate the MDR phenotype and P-gp is the target of oxidative damage, the SP cell fraction decreased due to P-gp damage.

### Cytotoxicity of DOX-NPs/Ce6-MBs treatment

**Fig. 7:**
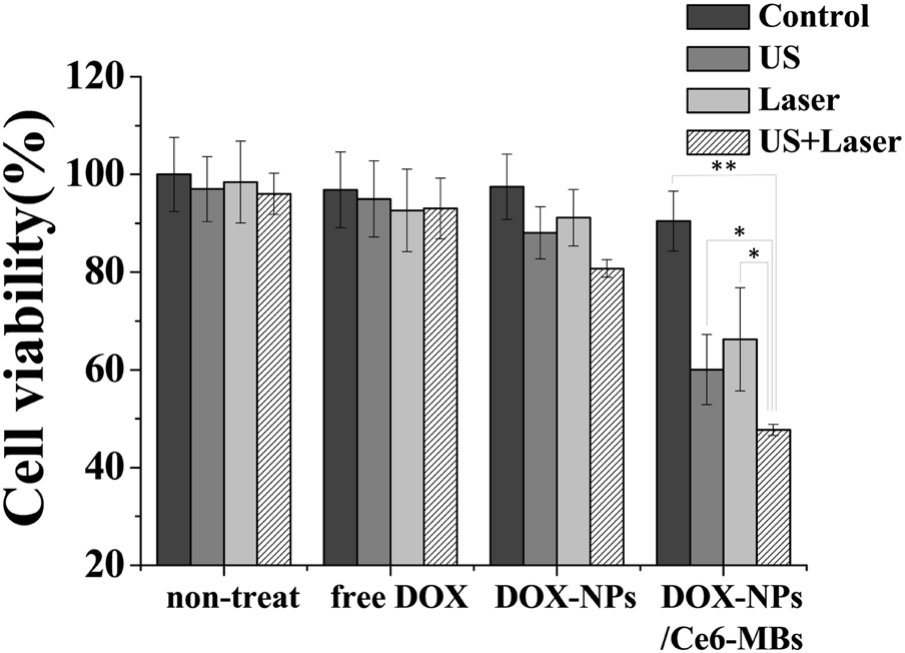
Cell viability assay. Cytotoxicity of DOX-NPs/Ce6-MBs complex and its incomplete comparison groups with and without US-, Laser-treated combination therapy. 671 nm laser was irradiated with 1.0 J/cm^2^, 50 mW per well. US was applied with a power of 0.2 W/cm^2^ and duty cycle of 50% for 5 s per well. Statistical analysis was performed using Student’s t-test (* P < 0.05, ** P < 0.01)

Based on the intracellular delivery (Fig. 3) and the ROS generation (Fig. 4) studies, the cell viability with combination of chemotherapy and PDT after delivery of the DOX-NPs/Ce6-MBs complex was evaluated in vitro (Fig. 7). First, exposure to US or/and laser did not result in any statistical difference between the treated groups and the control group, indicating that the US and laser themselves caused no cytotoxicity. Since the MCF-7/ADR cell line is DOX-resistant, the free DOX and DOX-NPs treated groups showed less toxicity compared to the DOX-NPs/Ce6-MBs-treated group (ANOVA, P < 0.01). For the DOX-NPs/Ce6-MBs group, there was significant cell death under the exposure of US because of the increased intracellular DOX and Ce6 delivery at US irradiation (Fig. 3). Also, the cells treated with DOX-NPs/Ce6-MBs and US showed statistically significant cytotoxicity because the sonoporation effect caused by the cavitation of microbubbles delivered sufficient amounts of DOX into the cells. The cells treated with DOX-NPs/Ce6-MBs and laser also showed statistically significant cytotoxicity because of ROS generation (Fig. 4). Ultimately, the DOX-NPs/Ce6-MBs showed the highest cytotoxic effect when both of US and laser were applied at the same time. The cytotoxicity results indicate that the chemo-PDT combination therapy was more efficient than any single therapy against multidrug resistant cancer cells.

## Discussion

In this study, we developed DOX-NPs/Ce6-MBs to deliver both anticancer drugs and photosensitizer simultaneously to overcome the limitation of multi-drug resistance in chemotherapy. In DOX-NPs/Ce6-MBs, the photosensitizer was loaded into the microbubble and the anticancer agent was encapsulated into the nanoparticles conjugated onto the surface of the microbubble. As illustrated in Scheme 1, the mechanisms of the DOX-NPs/Ce6-MBs to overcome the anticancer resistance can be divided into three steps: (1) the efficient intracellular delivery of two drugs by the sonoporation effect due to the cavitation of microbubbles under the exposure of US, (2) efflux receptor damage caused by ROS generated under laser irradiation, and (3) overcoming anticancer resistance by high intracellular concentration of anticancer drugs by decreased efflux efficiency of anticancer drugs.

When DOX-NPs/Ce6-MBs are irradiated with US, jet streaming occurs in the surrounding media due to the cavitation of microbubbles, which temporarily increases the permeability of the cell membrane, thereby transferring DOX-NPs and Ce6 into the cells effectively by the sonoporation effect [21, 22]. Sonoporation is a phenomenon that forms temporary pores in cell membranes by the jet stream generated as the microbubbles burst. Enhanced drug delivery using sonoporation effect was first reported by Tachibana et al. [23], and a lot of researches applied sonoporation in drug and gene delivery for increased delivery efficiency [13]. The blood–brain barrier (BBB) restricts the entry of most macromolecules to the brain from the blood stream due to tight junctions; however, the BBB can be disrupted with sonoporation effect of ultrasound and microbubbles [24, 25]. Therefore, the sonoporation effect due to the cavitation of microbubbles in the DOX-NPs/Ce6-MBs complex could effectively deliver DOX and Ce6 into the anticancer cells at US irradiation [26]. Moreover, after the cavitation of microbubbles, lipids, which are components of the microbubbles, were self-assembled into liposomes or micelles, and the hydrophobic Ce6 agent were loaded into self-assembled liposomes or micelles and delivered into cells [12, 16] As shown in the cellular uptake data (Fig. 3), we confirmed that DOX-NPs/Ce6-MBs were efficiently transferred into cancer cells under the exposure of US. DOX-NPs and Ce6-liposomes enter cells via sonoporation with US and can then induce more cell apoptosis by high cellular uptake.

DOX released from the DOX-NPs suppresses cell division by intercalating into the DNA of the nucleus and Ce6 with laser irradiation generates ROS, which induces cell death [27, 28]. The DCFDA assay data showed the increase in ROS generation due to the efficient delivery of Ce6 by the sonoporation effect when the cells were laser-irradiated (Fig. 4). Consequently, the DOX-NPs/Ce6-MBs effectively induced cancer cell death by chemotherapy and PDT in chemoresistant cancer cells. As a result, the combination of chemotherapy and PDT maximized the therapeutic effect, increased the tumor cell apoptosis by ROS generation, and increased drug accumulation [13]. Furthermore, ROS generated by Ce6 under laser irradiation inside cells damages nuclear DNA and intracellular molecules, including membrane proteins or lipids [29]. According to previous studies, increases in ROS non-selectively attack membrane proteins, including efflux pumps [1, 30]. In this process, proteins that are necessary for the survival of cancer cells are also damaged or suppressed by ROS generation. Moreover, when ROS is generated in mitochondria, the ROS can oxidize NADH into NAD^+^ directly. As a result, ATP cannot be synthesized by ATP synthase and the efflux pump become dysfunctional due to the lack of energy supply [31]. Therefore, we confirmed the effect of ROS on DOX retention based on the assumption that ROS will affect not only cell cytotoxicity but also efflux pumps (Fig. 5) which is a characteristic of MDR. When verapamil inhibited P-glycoprotein, DOX remained in the cytoplasm and a similar pattern was observed when the laser was applied to DOX-NPs/Ce6-MBs. This result suggests that the production of ROS inhibited the drug efflux, thereby further synergizing the chemotherapeutic effect of DOX.

Side population cells, also known as cancer stem cells (CSCs), are a small sub-population of cells in tumor that possess the capacity to tumor development, metastasis, and recurrence [32–34]. It has been determined that the failure of cancer treatment is due to the persistence of CSCs that evade the treatment regimen. Also, these SP cells overexpress the adenosine triphosphatase binding cassette (ABC) transporters, which contribute to multidrug resistance and the high survival rate of the cancer cells [35]. Owing to the existence of ABC transporters, Hoechst 33342 dye can be pumped out as a substrate, serving as the basis of side population assay. Due to these characteristics, the existence of SP cells is currently regarded as a major challenge for tumor treatment. Therefore, it is critical to develop effective strategies to eliminate SP population. Our data shows when laser and ultrasound were irradiated together, the SP cell fraction dramatically decreased to 0.93% showing that our strategies could effectively eliminate SP population and reduce the chance of cancer recurrence (Fig. 6). Figure 2 shows that MCF-7/ADR cells are resistant to DOX because of ABCB1overexpression, however, ABCB1 is not associated with Ce6 [36]. Therefore, it was found that the SP cell fraction could be reduced by effectively generating ROS and arresting the efflux pump receptors with laser irradiation. The cell death from chemotherapy with DOX and PDT by Ce6 with laser irradiation was confirmed by the in vitro cell cytotoxicity assay (Fig. 7). The data in Fig. 7 show that the combination of chemotherapy and PDT produced the best cytotoxicity. As a result, synergistic anticancer treatment outcomes were obtained in anticancer resistant MCF-7/ADR cells through chemo- and photodynamic combination therapy. Therefore, we suggest that the DOX-NPs/Ce6-MBs we developed have excellent therapeutic effects in MDR cells.

## Conclusion

In conclusion, our study demonstrated an effective strategy for cancer therapy using US and PDT to overcome MDR. The developed DOX-NPs/Ce6-MBs can deliver DOX and Ce6 into cancer cells with US irradiation. Therefore, the effect of chemotherapy can be enhanced by increasing the intracellular concentration of DOX. In addition, Ce6 delivered into the cytoplasm generates ROS with laser irradiation, leading to the PDT effect, which inhibits the effusion of DOX by damaging the efflux pumps, the main characteristic of MDR cells. As a result, high intracellular DOX concentrations can be maintained and finally induce cell apoptosis. Therefore, we suggest that DOX-NPs/Ce6-MBs with US and laser irradiation can be a useful method to overcome the limitations of chemotherapy in MDR cancer cells.

## Data Availability

The datasets used and/or analysed during the current study are available from the corresponding author on reasonable request.
